# Impact of efforts to prevent maternal deaths due to obstetric hemorrhage on trends in epidemiology and management of severe postpartum hemorrhage in Japan: a nationwide retrospective study

**DOI:** 10.1186/s12884-022-04824-7

**Published:** 2022-06-17

**Authors:** Akihiko Ueda, Baku Nakakita, Yoshitsugu Chigusa, Haruta Mogami, Shosuke Ohtera, Genta Kato, Masaki Mandai, Eiji Kondoh

**Affiliations:** 1 Department of Gynecology and Obstetrics, Kyoto University, 54 Shogoin Kawahara-cho, Sakyo, Kyoto, 606-8507 Japan; 2 Division of Medical Information Technology and Administration Planning, Kyoto University Hospital/National Institute of Public Health, Kyoto, Japan; 3 Solutions Center for Health Insurance Claims, Kyoto University Hospital, Kyoto, Japan

**Keywords:** Arterial embolization, Atonic hemorrhage, Blood transfusion, Hysterectomy, Maternal mortality, Postpartum hemorrhage, Red blood cell, Fresh frozen plasma, Uterine balloon tamponade

## Abstract

**Background:**

The Japan Society of Obstetrics and Gynecology and the Japan Association of Obstetricians and Gynecologists have issued the guidelines and recommendations on postpartum hemorrhage since 2010 and have been conducted widespread educational activities from 2012. The aim of this study was to investigate the impact of these efforts by the Societies to prevent maternal deaths due to obstetric hemorrhage on trends in epidemiology and management of severe postpartum hemorrhage in Japan.

**Methods:**

A national retrospective cohort study was conducted using the national database of health insurance claims for the period 2012 and 2018. The subjects were all insured women who received a blood transfusion for postpartum hemorrhage. The primary endpoints of this study were hysterectomy and maternal mortality. The etiology of hemorrhage, treatment facility, type of procedure, and blood transfusion volume were tabulated.

**Results:**

Women with postpartum hemorrhage that underwent transfusion increased from 3.5 to 5.5 per 1000 deliveries between 2012 and 2018. The most common cause of postpartum hemorrhage was atonic hemorrhage. After insurance coverage in 2013, the intrauterine balloon tamponade use increased to 20.3% of postpartum hemorrhages treated with transfusion in 2018, while the proportion of hysterectomy was decreased from 7.6% (2013–2015) to 6.4% (2016–2018) (*p* < 0.0001). The proportion of postpartum hemorrhage in maternal deaths decreased from 21.1% (2013–2015) to 14.1% (2016–2018) per all maternal deaths cases (*p* = 0.14). Cases with postpartum hemorrhage managed in large referral hospitals was increased (65.9% in 2012 to 70.4% in 2018) during the study period (*p* < 0.0001).

**Conclusions:**

The efforts by the Societies to prevent maternal mortality due to obstetric hemorrhage resulted in a significant decrease in the frequency of hysterectomies and a downward trend in maternal mortality due to obstetric hemorrhage.

**Supplementary Information:**

The online version contains supplementary material available at 10.1186/s12884-022-04824-7.

## Background

Postpartum hemorrhage (PPH) is a leading cause of pregnancy-related mortality worldwide [1]. Although PPH prevention and management improvements have reduced the rate of maternal mortality, the incidence of PPH increased in several developed countries during the 1990s and 2000s [2–4].

In Japan, unlike other developed countries, PPH had long been the leading cause of maternal death, accounting for 30% of maternal deaths. This is probably due to Japan’s health care system, in which birthplace is not fully centralized, with more than 2500 facilities managing approximately one million deliveries per year, and almost half of these deliveries are managed in small-scale obstetric facilities [5]. Obstetrical hemorrhage requires special care in cooperation with a higher-level facility or other departments. Therefore, the Japanese Clinical Practice Guide for Critical Obstetrical Hemorrhage was first issued from Japan Society of Obstetrics and Gynecology (JSOG) in 2010 [6]. Moreover, the Japan Association of Obstetricians and Gynecologists (JAOG) launched a registration system for maternal deaths and the Maternal Death Exploratory Committee in 2010. The committee conducts a causal analysis of each maternal death and publishes clinical and practical recommendations every year, with the aim of improving the quality of obstetric care. These clinical practice guides and recommendations include 1) blood transfusion of red blood cells (RBCs) and fresh frozen plasma (FFP) at a ratio of 1:1, 2) introduction of intrauterine balloon tamponade, 3) early decision to transfer severe PPH cases to large centers, and 4) early introduction of blood transfusion based on shock index [7]. Moreover educational lectures and simulation programs by JSOG and JAOG have been widely conducted from 2012. However, the impact of these efforts on trends in epidemiology and management of severe PPH remain unclear.

The present study aimed to investigate the impact of the efforts by the Societies (JSOG and JAOG) to prevent maternal deaths due to obstetric hemorrhage on trends in epidemiology and management of severe PPH in Japan.

## Methods

### An overview of the study

This study was a nationwide retrospective cohort study using the National Database of Health Insurance Claims and Specific Health Checkups of Japan (NDB) for the period 2012 and 2018 to analyze the trend in epidemiology and management of severe PPH after the introduction of educational activities by JSOG and JAOG from 2012. The study period covered only after implementation of the Societies activities. This is because, although the NDB has been accumulated since 2008, it was not until 2012 that the penetration of electronic medical record systems reached more than 90% of all facilities [8]. The subjects were all insured women who received a blood transfusion for postpartum hemorrhage. The primary endpoints of this study were hysterectomy and maternal mortality. Details of NDB, patient identification, and statistical methods were described as follows.

### National database of health insurance claims

The NDB is the largest insured patient database in Japan, operated by the Ministry of Health, Labour and Welfare (MHLW) from 2008 [9]. The NDB includes the following data of each patient: age, sex, primary and secondary diagnoses, procedures performed, medications and devices used, mortality, and hospital type and locations. Diagnoses and complications are recorded using the International Classification of Diseases Tenth Revision (ICD-10) codes and corresponding MHLW disease or procedure codes. Physicians in any facility are required to describe the diagnosis and procedures accurately, as these records are directly linked to reimbursement for health care costs, which ensures the reliability of the NDB. The database contains no laboratory data or findings on obstetric and imaging findings, including the amount of bleeding or coagulation status. In the NDB data, hospitals are classified into two categories based on participation in the diagnosis procedure combination (DPC), a prospective payment system to control health care costs. DPC facilities contain approximately 1200 large referral hospitals, including all 82 academic hospitals in Japan, whereas non-DPC facilities contain relatively small-scale hospitals such as private clinics.

### Guidelines, recommendations, and educational activities by the Societies in Japan

Until 2010, there were no uniform treatment protocols among facilities, and the PPH management was left to the physicians' discretion. As a result, some PPH cases could not be saved due to the delay in the initial treatment. Therefore, the guidelines and recommendations on PPH published by JSOG and JAOG in 2010 aimed to standardize the initial treatment of PPH by establishing objective criteria for the initiation of blood transfusion and emergency transport based on vital signs and shock index (calculated by heart rate divided by systolic blood pressure) [6, 7]. The Maternal Death Exploratory Committee, which was established by JAOG, has been analyzing the causes of maternal deaths and making recommendations every year since 2010, with the aim of preventing maternal deaths. In order to implement these guidelines and recommendations in all facilities in Japan, the publication of the guidelines and recommendations on PPH have been distributed to all members of the Societies. In addition, the Societies have provided educational lectures repeatedly. Furthermore, the maternal emergency simulation program (Japan Maternal Emergency Life-saving course, J-MELS), which was established in 2015 by seven organizations including the Societies, offers a four-hour curriculum consisting of lectures and simulations that provide early perception and initial care of maternal emergencies, decision making for maternal transport, and basic lifesaving support procedures. J-MELS has been provided throughout Japan, and in 2018, more than 10,000 of approximately 40,000 obstetric caregivers participated the program [7, 10].

### Patient identification

We identified all women who were diagnosed with severe PPH between January 2012 and December 2018. Traditionally, PPH has been defined as ≥ 500 mL for vaginal delivery and ≥ 1000 mL or more for cesarean section, but in clinical practice, the 90th percentile of blood loss during delivery in the Japanese population is considered one of the indicators of PPH (e.g., ≥ 800 mL for vaginal delivery and ≥ 1500 mL for cesarean section for a singleton pregnancy) [6]. On the other hand, there is no clear definition of severe PPH. In the present study, severe PPH was defined as PPH that was treated with blood transfusion. To identify PPH, we collected PPH etiologies, including uterine atony, placenta previa, placental abruption, uterine rupture, uterine inversion, cervical laceration, vaginal hematoma, placenta accrete, retained placenta, amniotic fluid embolism, and multiple gestation (Table S1). Those with uterine atony plus another PPH code were considered as non-atonic and were subjected to the PPH code. Placenta previa accrete were diagnosed when both placenta previa and placenta accrete were present. Placenta previa was defined as placenta previa without placenta accreta, and placenta accreta was defined as placenta accreta without placenta previa. In cases of placental abruption, intrauterine fetal deaths were identified. We collected clinical characteristics including maternal age and mode of delivery (caesarean section and operative vaginal delivery).

### Primary and secondary endpoints

Primary endpoints were hysterectomy and in-hospital maternal mortality. Secondary endpoints were intrauterine balloon tamponade, arterial embolization, the volume of transfused RBCs, FFP, and platelets, and FFP/RBCs ratio. The mean volume of blood transfusion was calculated respectively in RBCs, FFP, and platelet transfusion cases. FFP/RBCs ratio was analyzed in cases with RBCs transfusion and included the cases without FFP transfusion. The claims were also analyzed separately for hospital types (DPC or non-DPC facilities) that provided blood transfusions for severe PPH.

### Statistical method

All analyses were performed using software JMP® Pro 15.1.0 (SAS Institute Inc, Cary, NC, USA). Data were compiled by year and maternal age, and the incidence of PPH was calculated from the total number of deliveries obtained from the Vital Statistics Survey by MHLW [11]. The total number of maternal deaths was obtained from the registry data of the Japan Maternal Death Exploratory Committee [12], since the causes of all maternal deaths are too diverse to be collected from ICD10-based NDB. We present the categorical variables as numbers and percentages; comparisons were made by chi-square test. Continuous variables were shown as means and standard deviations or medians and interquartile ranges; comparisons were made by the Mann–Whitney U test. Due to the regulation of MHLW, we cannot disclose data less than ten, and the related rows that counted backwards to reveal the data, thus showing as “– “ and “*” in the results, respectively. The significance level was set at *p* < 0.05.

## Results

### Patient characteristics

The prevalence and clinical characteristics of patients with severe PPH are shown in Table 1. During the study period, the number of PPH patients who underwent a blood transfusion per year significantly increased from 3668 (3.54 per 1000 deliveries) in 2012 to 5049 (5.50 per 1000 deliveries) in 2018 (Chi-square test, *p* < 0.0001). The mean age increased from 32.9 ± 5.3 years in 2012 to 33.6 ± 5.4 years in 2018 (Mann–Whitney U test, *p* < 0.0001). Approximately 60% of cases of severe PPH occurred in women aged 30–39 years. The age-adjusted severe PPH incidence per 1000 deliveries showed an increasing trend per year in all age groups. The frequency was the lowest in the 20–24 years of age group (2.96 per 1000 deliveries in 2018), and the incidence increased with increasing age in the 25 years and older age group (25–29 years: 3.80; 30–34 years: 4.82; 35–39 years: 7.63; 40–44 years: 12.25; 45 years or more: 27.73 per 1000 deliveries in 2018, respectively). Cesarean deliveries and operative vaginal deliveries accounted for about 40% and 10% of the cases, respectively.

**Table 1:** Prevalence and clinical characteristics of patients with severe PPH

	2012	2013	2014	2015	2016	2017	2018	Comparison between 2012 and 2018^a^
Total number of deliveries, *n*	1,037,231	1,029,816	1,003,539	1,005,677	976,978	946,065	918,397	-
Patients with severe PPH, *n* (rate per 1000 deliveries)	3668 (3.54)	4021 (3.90)	4182 (4.17)	4513 (4.49)	4767 (4.88)	5061 (5.35)	5049 (5.50)	< .0001
Age, *n* (rate per 1000 deliveries)	32.9 ± 5.3	33.0 ± 5.3	33.3 ± 5.3	33.4 ± 5.4	33.5 ± 5.3	33.3 ± 5.3	33.6 ± 5.4	< .0001^b^
≤ 19	30 (2.35)	31 (2.39)	33 (2.54)	35 (2.93)	33 (2.97)	32 (3.23)	34 (3.87)	.3232
20–24	203 (2.12)	219 (2.40)	190 (2.19)	242 (2.87)	220 (2.68)	254 (3.20)	228 (2.96)	.0135
25–29	712 (2.43)	744 (2.63)	762 (2.84)	760 (2.90)	823 (3.28)	880 (3.65)	888 (3.80)	< .0001
30–34	1217 (3.31)	1322 (3.62)	1354 (3.77)	1452 (3.98)	1528 (4.31)	1697 (4.91)	1614 (4.82)	< .0001
35–39	1150 (5.10)	1289 (5.61)	1369 (6.06)	1438 (6.30)	1544 (6.91)	1603 (7.39)	1611 (7.63)	< .0001
40–44	325 (7.73)	395 (8.49)	437 (8.81)	550 (10.46)	598 (11.18)	568 (10.90)	628 (12.25)	< .0001
45 ≤	31 (32.29)	21 (18.82)	37 (29.09)	36 (27.52)	21 (14.99)	27 (17.86)	46 (27.73)	.0247
Mode of delivery, *n* (% of patients with severe PPH)								
Cesarean delivery	1467 (40.0)	1541 (38.3)	1652 (39.5)	1698 (37.6)	1769 (37.1)	1742 (34.4)	1820 (36.0)	.0002
Operative vaginal delivery	303 (8.3)	393 (9.8)	381 (9.1)	466 (10.3)	516 (10.8)	563 (11.1)	587 (11.6)	< .0001
Etiology of PPH, *n* (rate per 1000 deliveries)								
Uterine atony	1347 (1.30)	1634 (1.59)	1652 (1.65)	1817 (1.81)	1973 (2.02)	2157 (2.28)	2257 (2.46)	< .0001
Placenta previa	467 (0.45)	440 (0.43)	462 (0.46)	471 (0.47)	513 (0.53)	512 (0.54)	528 (0.57)	.0001
Placenta accreta	466 (0.45)	559 (0.54)	585 (0.58)	650 (0.65)	696 (0.71)	780 (0.82)	830 (0.90)	< .0001
Placenta previa accreta	97 (0.09)	105 (0.10)	91 (0.09)	110 (0.11)	103 (0.11)	99 (0.10)	91 (0.10)	.6919
Retained placenta	58 (0.06)	75 (0.07)	103 (0.1)	130 (0.13)	122 (0.12)	134 (0.14)	151 (0.16)	< .0001
Placental abruption	635 (0.61)	613 (0.60)	666 (0.66)	675 (0.67)	622 (0.64)	598 (0.63)	528 (0.57)	.2857
Cases with IUFD	162 (0.16)	178 (0.17)	150 (0.15)	138 (0.14)	132 (0.14)	128 (0.14)	96 (0.10)	.0017
Uterine rupture	85 (0.08)	86 (0.08)	100 (0.10)	98 (0.10)	83 (0.08)	97 (0.10)	84 (0.09)	.4750
Uterine inversion	132 (0.13)	102 (0.10)	120 (0.12)	99 (0.10)	98 (0.10)	138 (0.15)	101 (0.11)	.2690
Cervical laceration	68 (0.07)	54 (0.05)	59 (0.06)	72 (0.07)	94 (0.10)	101 (0.11)	72 (0.08)	.2896
Vaginal hematoma	253 (0.24)	284 (0.28)	293 (0.29)	305 (0.30)	342 (0.35)	365 (0.39)	324 (0.35)	< .0001
Cervical laceration with vaginal hematoma	125 (0.12)	153 (0.15)	139 (0.14)	178 (0.18)	184 (0.19)	205 (0.22)	194 (0.21)	< .0001
Amniotic fluid embolism	38 (0.04)	37 (0.04)	44 (0.04)	52 (0.05)	52 (0.05)	57 (0.06)	42 (0.05)	.3209
Multiple gestation	141 (0.14)	175 (0.17)	173 (0.17)	178 (0.18)	218 (0.22)	228 (0.24)	252 (0.27)	< .0001

*IUFD *Intrauterine fetal death, *PPH P*ostpartum hemorrhage

^a^Comparisons of categorical variables were made by chi-square test; comparisons of continuous variables were made by the Mann–Whitney U test^b^

The most common cause of severe PPH was atonic hemorrhage, followed by placenta accreta, placental abruption, and placenta previa in 2018. During 2012 and 2018, incidence of uterine atony (1.30 to 2.46 per 1000 deliveries), placenta previa (0.45 to 0.57 per 1000 deliveries), placenta accreta (0.45 to 0.90 per 1000 deliveries), cervical laceration and/or vaginal hematoma (0.43 to 0.64 per 1000 deliveries), and multiple gestation (0.14 to 0.27 per 1000 deliveries) were all significantly increased (Chi-square test, *p* ≤ 0.0001; Table 1), with no change in the incidence of placental abruption, uterine rupture, and uterine inversion. Table S2 describes the etiologies of severe PPH by maternal age. Interestingly, age-adjusted incidence per 1000 deliveries of uterine atony, placenta previa, placenta accreta, and placental abruption were the lowest in the 20–24 years of age, and the incidence increased with increasing age in the 25 years and older age group. In addition, age-adjusted incidence per 1000 deliveries of uterine rupture or inversion, cervical laceration, vaginal hematoma, and amniotic fluid embolism also increased with increasing age.

### Transfusion volume and hemostatic procedures

**Fig. 1:**
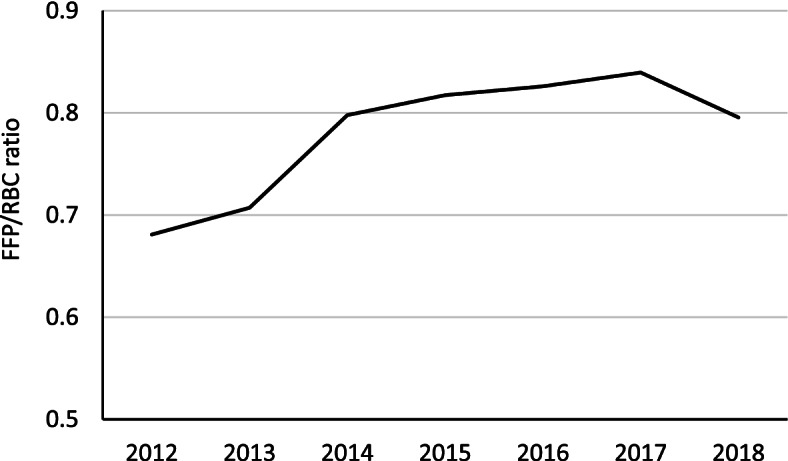
Trends in FFP/RBCs ratio per case. Annual change in FFP/RBCs ratio from 2012 to 2018. FFP: Fresh frozen plasma, RCB: red blood cells

Table 2 describes transfusion volumes and hemostatic procedures for patients with severe PPH. During the study period, the frequency of transfusion with FFP (from 63.0% to 70.5% of severe PPH, Chi-square test, *p* < 0.0001) were significantly increased, while RBCs (from 95.2% to 93.8% of severe PPH, Chi-square test, *p* = 0.0040) and platelet (from 14.1% to 11.0% of severe PPH, Chi-square test, *p* < 0.0001) were significantly decreased. Massive blood transfusion, which is defined as the transfusion of ≥ 10 RBCs units, was performed in 24.1% of all severe PPH cases in 2012, which decreased to 20.5% in 2018, though the incidence increased from 0.85 (883 cases among 1,037,231 deliveries) in 2012 to 1.13 (1035 cases among 918,397 deliveries) per 1000 deliveries in 2018 (Chi-square test, *p* < 0.0001). Intrauterine balloon tamponade for atonic hemorrhage was covered by insurance from 2013. After introducing intrauterine balloon tamponade, the number of intrauterine balloon tamponade use increased to 1025 cases (20.3% of severe PPH) in 2018. The mean volume of transfused RBCs (from 8.2 ± 7.5 to 7.2 ± 6.3 units, Mann–Whitney U test, *p* < 0.0001) and FFP (from 9.7 ± 10.0 to 8.7 ± 8.6 units, Mann–Whitney U test, *p* < 0.0001) were all significantly decreased during 2013 to 2018, while arterial embolization did not quite reach statistical significance (from 6.3% to 5.8% of severe PPH). The FFP/RBCs ratio per case increased significantly from 0.68 in 2012 to 0.80 in 2018 (Mann–Whitney U test, *p* < 0.0001; Fig. 1). The etiologies of severe PPH cases that underwent hemostatic procedures between 2013 and 2018 are described in Table S3. Intrauterine balloon tamponade was mainly performed in the cases with uterine atony (8.9% of severe PPH in 2017 and 2018) and placenta previa (3.3% of severe PPH). Arterial embolization was performed for uterine atony (2.3% of severe PPH) and placenta accreta (1.4% of severe PPH).

**Table 2:** Transfusion volumes and hemostatic procedures for patients with severe PPH

	2012	2013	2014	2015	2016	2017	2018
Patients with severe PPH, *n*	3668	4021	4182	4513	4767	5061	5049
The transfusion volume, unit							
Red blood cells, mean ± SD^a^	8.0 ± 7.7	8.2 ± 7.5	8.1 ± 8.1	8.3 ± 7.9	7.8 ± 6.9	7.5 ± 6.8	7.2 ± 6.3
Red blood cells, *n* (% of patients with severe PPH)							
0	176 (4.8)	195 (4.8)	230 (5.5)	269 (6.0)	279 (5.9)	282 (5.6)	315 (6.2)
1–9	2609 (71.1)	2757 (68.6)	2942 (70.3)	3060 (67.8)	3317 (69.6)	3646 (72.0)	3699 (73.3)
≥ 10	883 (24.1)	1069 (26.6)	1010 (24.2)	1184 (26.2)	1171 (24.6)	1133 (22.4)	1035 (20.5)
Fresh frozen plasma, mean ± SD^b^	9.5 ± 10.1	9.7 ± 10.0	9.9 ± 10.9	10.3 ± 10.7	9.6 ± 9.9	9.3 ± 9.7	8.7 ± 8.6
Fresh frozen plasma, n (% of patients with severe PPH)							
0	1358 (37.0)	1352 (33.6)	1257 (30.1)	1294 (28.7)	1343 (28.2)	1453 (28.7)	1491 (29.5)
1–9	1566 (42.7)	1733 (43.1)	1904 (45.5)	2044 (45.3)	2285 (47.9)	2427 (48.0)	2506 (49.6)
≥ 10	744 (20.3)	936 (23.3)	1021 (24.4)	1175 (26.0)	1139 (23.9)	1181 (23.3)	1052 (20.8)
Platelet, mean ± SD^c^	22.5 ± 16.7	21.9 ± 15.4	22.6 ± 17.7	22.5 ± 16.1	21.6 ± 16.8	22.0 ± 16.2	21.6 ± 16.5
Platelet, *n* (% of patients with severe PPH)							
0	3150 (85.9)	3452 (85.8)	3620 (86.6)	3870 (85.8)	4173 (87.5)	4473 (88.4)	4493 (89.0)
1–10	171 (4.7)	193 (4.8)	180 (4.3)	208 (4.6)	193 (4.0)	212 (4.2)	185 (3.7)
> 10	347 (9.5)	376 (9.4)	382 (9.1)	435 (9.6)	401 (8.4)	376 (7.4)	371 (7.3)
Hemostatic procedures, n (% of patients with severe PPH)							
Intrauterine balloon tamponade^d^		98 (2.4)	331 (7.9)	604 (13.4)	717 (15.0)	875 (17.3)	1025 (20.3)
Arterial embolization	174 (4.7)	255 (6.3)	300 (7.2)	319 (7.1)	324 (6.8)	294 (5.8)	291 (5.8)
Hysterectomy	320 (8.7)	347 (8.6)	291 (7.0)	331 (7.3)	323 (6.8)	311 (6.1)	313 (6.2)

*PPH* Postpartum hemorrhage, *SD* Standard deviation

^a^Mean amount of transfused red blood cells in patients that performed transfusion

^b^Mean amount of transfused fresh frozen plasma in patients that performed transfusion

^c^Mean amount of transfused platelet in patients that performed transfusion

^d^Intrauterine balloon tamponade for abdominal hemorrhage were covered by insurance and thus counted since 2013

### Hysterectomy and maternal mortality of patients with severe PPH

Table 3 shows the hysterectomy and maternal mortality of patients with severe PPH. The proportion of hysterectomy was significantly decreased from 7.6% of severe PPH between 2013 and 2015 to 6.4% of severe PPH between 2016 and 2018 (Chi-square test, *p* < 0.0001). The main causes of hysterectomy were placenta accreta (1.9% of severe PPH) and uterine atony (1.3% of severe PPH). Among hysterectomy cases, uterine atony decreased from 2.0% of severe PPH (2013–2014) to 1.3% (2017–2018) (Chi-square test, *p* = 0.0001; Table S3). The proportion of maternal mortality due to severe PPH was decreased from 21.1% of maternal deaths (0.92 per 100,000 births) between 2013 and 2015 to 14.1% of maternal deaths (0.63 per 100,000 births) between 2016 and 2018, although the analysis did not quite reach statistical significance (Chi-square test, *p* = 0.14). It was noteworthy that approximately 90% of maternal deaths occurred in non-DPC facilities.

**Table 3:** Hysterectomy and maternal mortality of patients with severe postpartum hemorrhage

	2013–2015	2016–2018	*p* value^b^
Patient with severe PPH, *n*	12,716	14,877	-
Total maternal deaths^a^, *n*	133	128	-
Hysterectomy, n (% of patients with severe PPH)	969 (7.6)	947 (6.4)	< .0001
Maternal mortality due to severe PPH, *n*	28	18	-
Case, per 100,000 deliveries	0.92	0.63	-
Case, % of total maternal deaths^a^	21.1	14.1	.1384
Case in non-DPC facilities, *n*	27	16	-

*PPH* Postpartum hemorrhage

^a^The total number of maternal deaths was obtained from the registry data of the Japan Maternal Death Exploratory Committee [10]

^b^Comparisons were made by chi-square test

### Type of obstetric facilities that provided blood transfusion for PPH

**Fig. 2:**
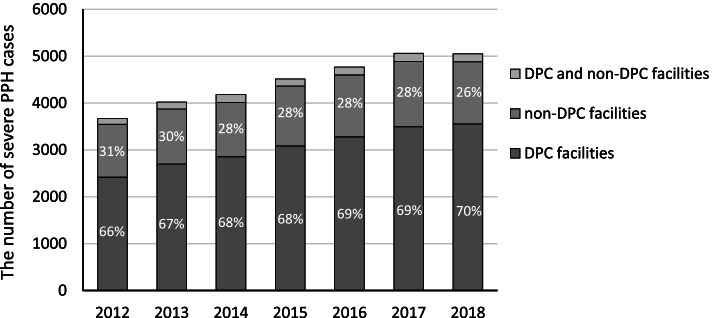
Type of obstetric facilities that provided blood transfusion for PPH. Obstetric facilities from 2012 to 2018 were divided by facility category. When a patient received a blood transfusion at both DPC and non-DPC facilities, it means the patient was transported from non-DPC to DPC facility. DPC: diagnosis procedure combination, PPH: postpartum hemorrhage. DPC facilities contain large referral hospitals, whereas non-DPC facilities contain relatively small-scale hospitals

The type and location of the treatment facilities are described in Fig. 2. Approximately 70% of PPH cases were treated in DPC facilities, while 30% of those were treated in non-DPC facilities or the cases of treated both in non-DPC and DPC facilities. The proportion of PPH cases managed in DPC facilities was significantly increased from 2012 to 2018 (65.9% to 70.4% of severe PPH, Chi-square test, *p* < 0.0001), whereas it decreased significantly in non-DPC facilities (30.8% to 26.3% of severe PPH, Chi-square test, *p* < 0.0001).

The etiologies of severe PPH cases that underwent hemostatic procedures are described in Table S4. In DPC facilities, frequency of uterine atony, placenta accreta, and multiple gestation were significantly increased from 2012 to 2018 (29.4% to 38.7%, 12.9% to 17.9%, and 4.2% to 6.0% of severe PPH managed in DPC facilities, respectively, *p* < 0.005), whereas that of placental abruption was decreased (22.8% to 12.3% of severe PPH managed in DPC facilities, Chi-square test, *p* < 0.0001). In non-DPC facilities, more than half of severe PPH cases were uterine atony. The frequency of uterine atony was significantly increased from 2012 to 2018 (51.9% to 60.2% of severe PPH managed in non-DPC facilities, Chi-square test, *p* < 0.0001), whereas that of placenta previa was decreased significantly (12.3% to 7.1% of severe PPH managed in non-DPC facilities, Chi-square test, *p* < 0.0001).

The frequency of blood transfusion and hemostatic treatments by the type of obstetric facility is shown in Table S5. The use of FFP was significantly increased in non-DPC facilities from 2012 to 2018 (50.2% to 73.2% of severe PPH managed in non-DPC facilities, Chi-square test, *p* < 0.0001). The use of intrauterine balloon tamponade increased in all types of facilities, and the frequency of hysterectomy decreased significantly from 2013 to 2018 in both DPC facilities (9.3% to 6.7%, Chi-square test, *p* = 0.0001) and non-DPC facilities (7.1% to 4.7%, Chi-square test, *p* = 0.01).

## Discussion

### Main findings

Analysis of the national database of health insurance claims in Japan revealed 7-year trends in epidemiology and management of severe PPH (2012–2018). The results showed that (1) the incidence of PPH treated with blood transfusion has increased, (2) the frequency of hysterectomies has decreased with a downward trend in maternal mortality, (3) the FFP/RBCs ratio and balloon tamponade usage have increased, and (4) the proportion of severe PPH managed in DPC facilities (large referral hospitals) has increased.

### Interpretation

Early detection and appropriate initial treatment of severe PPH without delay is crucial in preventing maternal death. Before 2010, the need for blood transfusion or emergency maternal transport for PPH were usually determined by measured amount of blood loss, with little attention paid to vital signs. However, it was difficult to accurately measure the amount of blood loss after delivery, and initial response was often delayed, and there were some reports of cardiac arrest during maternal transport. In fact, the Maternal Death Exploratory Committee estimated that about half of the maternal deaths due to PPH could have been saved if appropriate measures had been taken [13]. Therefore, the Societies have been conducting educational activities for obstetric caregivers from all types of obstetric facilities to ensure that the standardized protocols such as the use of shock index as an objective indicator of initiating blood transfusion, the use of RBCs and FFP at a ratio of 1:1, the use of intrauterine balloon, and early decision to transfer severe PPH cases to large facilities are uniformly implemented at all facilities throughout Japan. These efforts may have led to significant increases in the frequency of FFP/RBCs ratios in primary facilities, intrauterine balloon tamponade use, and increased proportion of cases managed in large referral hospitals, as a result, to a reduction in the frequency of hysterectomies and a downward trend in maternal mortality.

This study revealed that both PPH treated with transfusion and the proportion of massive transfusion have increased in the last seven years. The significant increase in the number of severe PPH cases, including massive transfusion cases, may be associated with higher maternal age. In an age-adjusted comparison of the frequency of severe PPH, the severe PPH frequency was the lowest in the age group of 20–24 years, and the risk was more than twice as high in the age group of 35 years and older. Since the previous report also identified age over 35 years as a risk for severe PPH [14], it is essential to recognize that maternal age is an significant risk factor for severe PPH. Another possible reason for the increase in PPH treated with blood transfusion is the early introduction of blood transfusion in PPH cases, as recommended by JSOG and JAOG. So far, there are no clear criteria for initiation of transfusion, which is determined based on the patient's condition and clinical data [15–17]. Nevertheless, the recommendation for early introduction of transfusion may have lowered the hurdle for indication of transfusion and increased the number of PPH cases treated with transfusion. In a strict sense, it is difficult to assess the trend in patients with truly severe PPH because the database used in this study does not include detailed data on blood loss and laboratory results. However, a 7-year study of severe PPH patients nationwide showed a steady decline in maternal deaths and hysterectomies despite an increase in the number of PPH patients treated with transfusions as well as massive transfusions, suggesting that the JSOG and JAOG efforts are appropriate in a unique country where small obstetric facilities handle nearly half of all deliveries.

For facilities that treated PPH, the frequency of FFP transfusion and balloon tamponade usage in all types of facilities was increased, suggesting that treatment uniformity was achieved in all centers. However, given that most maternal deaths due to severe PPH occur in small facilities, there remains the unresolved issue of standardizing treatment for refractory PPH according to facilities, regions, and countries with diverse medical resources [18–20]. The national database of health insurance claims used in this study contains data on almost all of the nation’s population, making it possible to compare facilities and regions with various levels of medical resources. Further analysis of this data may provide new insights into establishing an evidence-based bundle for refractory PPH and reducing preventable maternal bleeding-related deaths, depending on the realities of health care that can be provided.

### Strengths and limitations

The strength of the study is that we analyzed a national database containing all birthing facilities in Japan for the periods as long as seven years. In addition, academic guidelines from two Japanese societies have been recognized and widely accepted as standard recommendations in Japan to reduce maternal mortality from obstetric hemorrhage. We believe that the widespread acceptance of academic guidelines on postpartum hemorrhage by two Japanese societies and the gradual decrease in maternal mortality as well as frequency of hysterectomy due to postpartum hemorrhage during the same period are compelling results. Despite these strengths, since this study is not a prospective study with intervention, it does not directly prove that the efforts by JSOG and JAOG have reduced hysterectomies and maternal deaths due to obstetric hemorrhage. In addition, since more than 10% of the health insurance claims were not registered electronically and not included in the NDB prior to 2011, this study was not able to compare epidemiological changes in PPH before and after the implementation of the JSOG and JAOG initiatives.

## Conclusion

Even in countries where obstetric facilities of various sizes are in disarray, widespread dissemination of management guidelines for severe PPH based on the objective index of shock index may contribute to the reduction of hysterectomies and maternal deaths.

## Supplementary Information

**Additional file 1:** **Supplementary Table 1.** The International Classification of Diseases (Tenth Revision) andcorresponding MHLW codes for etiologies of PPH. **Supplementary** **Table 2.** Etiologiesof patients with severe postpartum hemorrhage by maternal age. **Supplementary** **Table 3. **Etiologiesof severe PPH cases that underwent hemostatic procedures between 2013 and 2018. **Supplementary** **Table 4. **Etiologiesof severe PPH cases by the type of treatment facilities. **Supplementary** **Table 5. **The frequencyof blood transfusion and hemostatic treatments by the type of treatmentfacilities.  

## Data Availability

The datasets generated and/or analysed during the current study are not publicly available or cannot be made available upon request due to the restriction guidelines (Guidelines for the Provision of Anonymous Receipt Information and Anonymous Specified Health Examination Information, https://www.mhlw.go.jp/content/12400000/000678473.pdf) of the Ministry of Health, Labour and Welfare (Japan).
