# Supplementary material for: Impact of efforts to prevent maternal deaths due to obstetric hemorrhage on trends in epidemiology and management of severe postpartum hemorrhage in Japan: a nationwide retrospective study
Source: BMC Pregnancy Childbirth. 2022 Jun 17;22:496. doi: 10.1186/s12884-022-04824-7 (PMC9205073; doi:10.1186/s12884-022-04824-7)
Supplement: Supplementary file 1 — Additional file 1: Supplementary Table 1. The International Classification of Diseases (Tenth Revision) andcorresponding MHLW codes for etiologies of PPH. Supplementary Table 2. Etiologiesof patients with severe postpartum hemorrhage by maternal age. Supplementary Table 3. Etiologiesof severe PPH cases that underwent hemostatic procedures between 2013 and 2018. Supplementary Table 4. Etiologiesof severe PPH cases by the type of treatment facilities. Supplementary Table 5. The frequencyof blood transfusion and hemostatic treatments by the type of treatmentfacilities. [file 12884_2022_4824_MOESM1_ESM.doc]

**Supplementary Table 1.** The International Classification of Diseases (Tenth Revision) and corresponding MHLW codes for etiologies of PPH

| Etiologies of PPH | ICD-10 code | MHLW disease code |
| --- | --- | --- |
| Uterine atony | O72.1 | 8834172, 8834227, 8839870 |
| Placenta previa | O44.1 | 6410002, 6411001, 6411003, 6411004, 6411005, 6411006, 6411007, 6567002, 8834646, 8834647 |
| Placental abruption | O45.0, O45.9 | 6412001, 8846571 |
| Uterine rupture | O71.0, O71.1 | 6651001, 8839869, 8839880, 8839885 |
| Uterine inversion | O71.2 | 6217001, 6217002, 6217003, 8834263, 8839868 |
| Cervical laceration | O71.3, O71.4 | 6223005, 6653004, 6658001, 8833337 |
| Vaginal hematoma | O71.7 | 6245003, 6645001, 6659001, 8830824, 8832704, 8834050, 8834051, 8834054, 8834059, 8837499 |
| Placenta accrete | O72.0 | 6660002, 660007, 6670003, 6670006, 8845149, 8845170 |
| Retained placenta | O72.2 | 6660003, 6670005, 6671004 |
| Amniotic fluid embolism | O88.1 | 8840796 |
| Multiple gestation | O30.0, O30.1, O30.2, O30.8, O30.9 | 6510001, 6510003, 6510004, 6511001, 6511002, 6512001, 6512002, 6518001, 6518002, 6519001, 6519002, 7623002, 8845517, 8845518, 8845591 |

MHLW: the Ministry of Health, Labour and Welfare, PPH: postpartum hemorrahge.

**Supplementary Table 2.** Etiologies of patients with severe postpartum hemorrhage by maternal age

**Uterine atony, *n* (rate per 1000 deliveries)**

| Age, year | 2013–2015 | 2016–2018 |
| --- | --- | --- |
| ≤19 | 60 (1.58) | 49 (1.65) |
| 20–24 | 307 (1.17) | 351 (1.47) |
| 25–29 | 950 (1.17) | 1175 (1.62) |
| 30–34 | 1651 (1.52) | 2066 (2.00) |
| 35–39 | 1566 (2.29) | 1983 (3.04) |
| 40–44 | 540 (3.63) | 729 (4.65) |
| 45≤ | 29 (7.85) | 34 (7.44) |

**Placenta previa, *n* (rate per 1000 deliveries)**

| Age, year | 2013–2015 | 2016–2018 |
| --- | --- | --- |
| ≤19 | – (–) | – (–) |
| 20–24 | 32 (0.12) | 39 (0.16) |
| 25–29 | 169 (0.21) | 192 (0.26) |
| 30–34 | 420 (0.39) | 478 (0.46) |
| 35–39 | 543 (0.79) | 559 (0.86) |
| 40–44 | 193 (1.30) | 266 (1.70) |
| 45≤ | * (*) | * (*) |

**Placenta accreta, *n* (rate per 1000 deliveries)**

| Age, year | 2013–2015 | 2016–2018 |
| --- | --- | --- |
| ≤19 | 10 (0.26) | 12 (0.40) |
| 20–24 | 83 (0.32) | 68 (0.29) |
| 25–29 | 269 (0.33) | 328 (0.45) |
| 30–34 | 582 (0.53) | 752 (0.73) |
| 35–39 | 609 (0.89) | 786 (1.21) |
| 40–44 | 226 (1.52) | 345 (2.20) |
| 45≤ | 15 (4.06) | 15 (3.28) |

**Placental abruption, *n* (rate per 1000 deliveries)**

| Age, year | 2013–2015 | 2016–2018 |
| --- | --- | --- |
| ≤19 | * (*) | 12 (0.40) |
| 20–24 | 96 (0.37) | 93 (0.39) |
| 25–29 | 388 (0.48) | 310 (0.43) |
| 30–34 | 680 (0.62) | 622 (0.60) |
| 35–39 | 595 (0.87) | 548 (0.84) |
| 40–44 | 177 (1.19) | 151 (0.96) |
| 45≤ | – (–) | 12 (2.62) |

**Uterine rupture, *n* (rate per 1000 deliveries)**

| Age, year | 2013–2015 | 2016–2018 |
| --- | --- | --- |
| ≤19 | – (–) | – (–) |
| 20–24 | – (–) | – (–) |
| 25–29 | 29 (0.04) | 28 (0.04) |
| 30–34 | 77 (0.07) | 79 (0.08) |
| 35–39 | 127 (0.19) | 98 (0.15) |
| 40–44 | 43 (0.29) | 49 (0.31) |
| 45≤ | – (–) | – (–) |

**Uterine inversion, *n* (rate per 1000 deliveries)**

| Age, year | 2013–2015 | 2016–2018 |
| --- | --- | --- |
| ≤19 | – (–) | – (–) |
| 20–24 | 28 (0.11) | 27 (0.11) |
| 25–29 | 84 (0.10) | 83 (0.11) |
| 30–34 | 95 (0.09) | 115 (0.11) |
| 35–39 | 80 (0.12) | 85 (0.13) |
| 40–44 | 29 (0.20) | 24 (0.15) |
| 45≤ | – (–) | – (–) |

**Cervical laceration, *n* (rate per 1000 deliveries)**

| Age, year | 2013–2015 | 2016–2018 |
| --- | --- | --- |
| ≤19 | – (–) | – (–) |
| 20–24 | 10 (0.04) | 27 (0.11) |
| 25–29 | 41 (0.05) | 46 (0.06) |
| 30–34 | 66 (0.06) | 89 (0.09) |
| 35–39 | 53 (0.08) | 83 (0.13) |
| 40–44 | 13 (0.09) | * (*) |
| 45≤ | – (–) | – (–) |

**Vaginal hematoma, *n* (rate per 1000 deliveries)**

| Age, year | 2013–2015 | 2016–2018 |
| --- | --- | --- |
| ≤19 | – (–) | * (*) |
| 20–24 | 52 (0.20) | 60 (0.25) |
| 25–29 | 219 (0.27) | 282 (0.39) |
| 30–34 | 325 (0.30) | 341 (0.33) |
| 35–39 | 224 (0.33) | 264 (0.41) |
| 40–44 | 45 (0.30) | 59 (0.38) |
| 45≤ | * (*) | – (–) |

**Amniotic fluid embolism, *n* (rate per 1000 deliveries)**

| Age, year | 2013–2015 | 2016–2018 |
| --- | --- | --- |
| ≤19 | – (–) | – (–) |
| 20–24 | – (–) | – (–) |
| 25–29 | 13 (0.02) | 20 (0.03) |
| 30–34 | 41 (0.04) | 46 (0.04) |
| 35–39 | 60 (0.09) | 57 (0.09) |
| 40–44 | 16 (0.11) | 20 (0.13) |
| 45≤ | – (–) | – (–) |

**Multiple gestation, *n* (rate per 1000 deliveries)**

| Age, year | 2013–2015 | 2016–2018 |
| --- | --- | --- |
| ≤19 | – (–) | – (–) |
| 20–24 | 18 (0.07) | 34 (0.14) |
| 25–29 | 84 (0.10) | 109 (0.15) |
| 30–34 | 163 (0.15) | 234 (0.23) |
| 35–39 | 198 (0.29) | 228 (0.35) |
| 40–44 | 48 (0.32) | 85 (0.54) |
| 45≤ | * (*) | – (–) |

Due to the regulation of the Ministry of Health, Labour and Welfare, we cannot disclose data less than ten, and the related rows that counted backwards to reveal the data, thus showing as "-" and “*” in the results, respectively.

**Supplementary Table 3. Etiologies of severe PPH cases that underwent hemostatic procedures between 2013 and 2018.**

|  | 2013–2014 | 2015–2016 | 2017–2018 |
| --- | --- | --- | --- |
| Intrauterine balloon tamponade, *n* (% of patients with severe PPH) |  |  |  |
| Uterine atony | 194 (2.4) | 616 (6.6) | 896 (8.9) |
| Placenta previa | 75 (0.9) | 222 (2.4) | 333 (3.3) |
| Placenta accreta | 51 (0.6) | 185 (2.0) | 311 (3.1) |
| Placenta previa accreta | 13 (0.2) | 22 (0.2) | 40 (0.4) |
| Retained placenta | 12 (0.1) | 40 (0.4) | 61 (0.6) |
| Placental abruption | 42 (0.5) | 109 (1.2) | 106 (1.0) |
| Uterine inversion | 18 (0.2) | 32 (0.3) | 45 (0.4) |
| Amniotic fluid embolism | – (–) | 28 (0.3) | 34 (0.3) |
| Multiple gestation | 39 (0.5) | 119 (1.3) | 184 (1.8) |
| Arterial embolization, *n* (% of patients with severe PPH) |  |  |  |
| Uterine atony | 226 (2.8) | 246 (2.7) | 235 (2.3) |
| Placenta previa | 39 (0.5) | 53 (0.6) | 34 (0.3) |
| Placenta accreta | 116 (1.4) | 140 (1.5) | 144 (1.4) |
| Placenta previa accreta | 17 (0.2) | – (–) | 11 (0.1) |
| Retained placenta | 37 (0.5) | 41 (0.4) | 49 (0.5) |
| Placental abruption | 17 (0.2) | 23 (0.2) | 13 (0.1) |
| Vaginal hematoma | 59 (0.7) | 85 (0.9) | 65 (0.6) |
| Multiple gestation | 30 (0.4) | 35 (0.4) | 41 (0.4) |
| Hysterectomy, *n* (% of patients with severe PPH) |  |  |  |
| Uterine atony | 168 (2.0) | 146 (1.6) | 131 (1.3) |
| Placenta previa | 72 (0.9) | 72 (0.8) | 65 (0.6) |
| Placenta accreta | 146 (1.8) | 173 (1.9) | 190 (1.9) |
| Placenta previa accreta | 129 (1.6) | 140 (1.5) | 125 (1.2) |
| Retained placenta | 15 (0.2) | 22 (0.2) | 18 (0.2) |
| Placental abruption | 40 (0.5) | 33 (0.4) | 30 (0.3) |
| Uterine rupture | 39 (0.5) | 39 (0.4) | 37 (0.4) |
| Uterine inversion | 12 (0.1) | 13 (0.1) | 11 (0.1) |
| Amniotic fluid embolism | 23 (0.3) | 32 (0.3) | 33 (0.3) |
| Multiple gestation | 32 (0.4) | 39 (0.4) | 40 (0.4) |

PPH, postpartum hemorrhage.

Due to the regulation of the Ministry of Health, Labour and Welfare, we cannot disclose data less than ten, thus showing as "–" in the results.

**Supplementary Table 4.** Etiologies of severe PPH cases by the type of treatment facilities.

|  | 2012 | 2013 | 2014 | 2015 | 2016 | 2017 | 2018 |
| --- | --- | --- | --- | --- | --- | --- | --- |
| DPC facilities, *n* (% of patients with severe PPH managed in DPC facilities) |  |  |  |  |  |  |  |
| Uterine atony | 711 (29.4) | 926 (34.3) | 939 (32.9) | 1043 (33.8) | 1159 (35.3) | 1268 (36.3) | 1376 (38.7) |
| Placenta previa | 321 (13.3) | 307 (11.4) | 348 (12.2) | 334 (10.8) | 383 (11.7) | 413 (11.8) | 431 (12.1) |
| Placenta accreta | 313 (12.9) | 365 (13.5) | 421 (14.8) | 472 (15.3) | 521 (15.9) | 567 (16.2) | 637 (17.9) |
| Placenta previa accreta | 53 (2.2) | 59 (2.2) | 48 (1.7) | 54 (1.8) | 62 (1.9) | 54 (1.5) | 57 (1.6) |
| Retained placenta | 49 (2.0) | 69 (2.6) | 89 (3.1) | 122 (4.0) | 100 (3.0) | 116 (3.3) | 130 (3.7) |
| Placental abruption | 551 (22.8) | 525 (19.5) | 551 (19.3) | 585 (19.0) | 527 (16.1) | 506 (14.5) | 438 (12.3) |
| Intrauterine fetal death | 148 (6.1) | 159 (5.9) | 131 (4.6) | 127 (4.1) | 119 (3.6) | 115 (3.3) | 82 (2.3) |
| Uterine rupture | 62 (2.6) | 71 (2.6) | 74 (2.6) | 73 (2.4) | 62 (1.9) | 77 (2.2) | 63 (1.8) |
| Uterine inversion | 105 (4.3) | 77 (2.9) | 96 (3.4) | 75 (2.4) | 81 (2.5) | 120 (3.4) | 79 (2.2) |
| Cervical laceration | – (–) | – (–) | – (–) | – (–) | – (–) | – (–) | – (–) |
| Vaginal hematoma | 191 (7.9) | 223 (8.3) | 230 (8.1) | 240 (7.8) | 285 (8.7) | 273 (7.8) | 254 (7.1) |
| Amniotic fluid embolism | 26 (1.1) | 25 (0.9) | 27 (0.9) | 31 (1.0) | 29 (0.9) | 38 (1.1) | 27 (0.8) |
| Multiple gestation | 101 (4.2) | 133 (4.9) | 123 (4.3) | 148 (4.8) | 190 (5.8) | 192 (5.5) | 213 (6.0) |
| Non-DPC facilities, *n* (% of patients with severe PPH managed in non-DPC facilities) |  |  |  |  |  |  |  |
| Uterine atony | 585 (51.9) | 639 (54.4) | 634 (54.7) | 718 (56.3) | 747 (56.6) | 815 (58.5) | 800 (60.2) |
| Placenta previa | 139 (12.3) | 128 (10.9) | 111 (9.6) | 129 (10.1) | 125 (9.5) | 97 (7.0) | 94 (7.1) |
| Placenta accreta | 133 (11.8) | 162 (13.8) | 129 (11.1) | 148 (11.6) | 151 (11.4) | 176 (12.6) | 154 (11.6) |
| Placenta previa accreta | 43 (3.8) | 46 (3.9) | 42 (3.6) | 53 (4.2) | 39 (3.0) | 40 (2.9) | 31 (2.3) |
| Retained placenta | – (–) | – (–) | 10 (0.9) | – (–) | 11 (0.8) | – (–) | 13 (1.0) |
| Placental abruption | 77 (6.8) | 83 (7.1) | 102 (8.8) | 82 (6.4) | 82 (6.2) | 87 (6.3) | 85 (6.4) |
| Intrauterine fetal death | 12 (1.1) | 17 (1.4) | 19 (1.6) | 11 (0.9) | – (–) | 12 (0.9) | 13 (1.0) |
| Uterine rupture | 17 (1.5) | 13 (1.1) | 20 (1.7) | 16 (1.3) | 15 (1.1) | – (–) | 18 (1.4) |
| Uterine inversion | 23 (2.0) | 18 (1.5) | 21 (1.8) | 17 (1.3) | 14 (1.1) | 14 (1.0) | 17 (1.3) |
| Cervical laceration | 61 (5.4) | 49 (4.2) | 55 (4.7) | 64 (5.0) | 88 (6.7) | 94 (6.8) | 68 (5.1) |
| Vaginal hematoma | 45 (4.0) | 50 (4.3) | 49 (4.2) | 50 (3.9) | 44 (3.3) | 70 (5.0) | 61 (4.6) |
| Amniotic fluid embolism | 11 (1.0) | – (–) | 15 (1.3) | 18 (1.4) | 14 (1.1) | 18 (1.3) | 13 (1.0) |
| Multiple gestation | 40 (3.5) | 39 (3.3) | 45 (3.9) | 28 (2.2) | 24 (1.8) | 35 (2.5) | 34 (2.6) |

PPH, postpartum hemorrhage.

Due to the regulation of the Ministry of Health, Labour and Welfare, we cannot disclose data less than ten, thus showing as "–" in the results.

**Supplementary Table 5.** The frequency of blood transfusion and hemostatic treatments by the type of treatment facilities.

|  | 2012 | 2013 | 2014 | 2015 | 2016 | 2017 | 2018 |
| --- | --- | --- | --- | --- | --- | --- | --- |
| DPC facilities |  |  |  |  |  |  |  |
| Cases, n (% of severe PPH) | 2417 (65.9) | 2697 (67.1) | 2854 (68.2) | 3085 (68.4) | 3279 (68.8) | 3494 (69.0) | 3553 (70.4) |
| Frequency of blood transfusion,  n (% of severe PPH managed in DPC facilities) |  |  |  |  |  |  |  |
| Red blood cells | 2312 (95.7) | 2582 (95.7) | 2749 (96.3) | 2962 (96) | 3129 (95.4) | 3352 (95.9) | 3413 (96.1) |
| Fresh frozen plasma | 1635 (67.6) | 1831 (67.9) | 1998 (70.0) | 2200 (71.3) | 2316 (70.6) | 2418 (69.2) | 2425 (68.3) |
| Platelet | 408 (16.9) | 443 (16.4) | 440 (15.4) | 512 (16.6) | 441 (13.4) | 429 (12.3) | 445 (12.5) |
| Amount of blood transfusion, mean ± SD |  |  |  |  |  |  |  |
| Red blood cells* | 8.3 ± 7.9 | 8.5 ± 7.7 | 8.3 ± 8.0 | 8.5 ± 7.9 | 8.0 ± 6.9 | 7.5 ± 6.5 | 7.3 ± 6.6 |
| Fresh frozen plasma† | 9.9 ± 10.3 | 10.4 ± 10.6 | 10.6 ± 10.8 | 11.0 ± 10.9 | 10.2 ± 10.1 | 9.7 ± 9.2 | 9.2 ± 8.8 |
| Platelet‡ | 22.6 ± 16.4 | 21.9 ± 15.5 | 22.6 ± 17.4 | 21.9 ± 15.0 | 20.9 ± 15.1 | 21.4 ± 15.7 | 21.1 ± 16.8 |
| Hemostatic intervention, n (% of severe PPH managed in DPC facilities) |  |  |  |  |  |  |  |
| Intrauterine balloon tamponade§ |  | 67 (2.5) | 256 (9.0) | 451 (14.6) | 512 (15.6) | 636 (18.2) | 772 (21.7) |
| Arterial embolization | 148 (6.1) | 214 (7.9) | 247 (8.7) | 270 (8.8) | 265 (8.1) | 236 (6.8) | 243 (6.8) |
| Hysterectomy | 229 (9.5) | 252 (9.3) | 195 (6.8) | 224 (7.3) | 236 (7.2) | 215 (6.2) | 238 (6.7) |
| Non-DPC facilities |  |  |  |  |  |  |  |
| Cases, n (% of severe PPH) | 1128 (30.8) | 1174 (29.2) | 1159 (27.7) | 1275 (28.3) | 1319 (27.7) | 1392 (27.5) | 1330 (26.3) |
| Frequency of blood transfusion,  n (% of severe PPH managed in non-DPC facilities) |  |  |  |  |  |  |  |
| Red blood cells | 1057 (93.7) | 1094 (93.2) | 1035 (89.3) | 1129 (88.5) | 1190 (90.2) | 1252 (89.9) | 1157 (87.0) |
| Fresh frozen plasma | 566 (50.2) | 706 (60.1) | 771 (66.5) | 874 (68.5) | 946 (71.7) | 1026 (73.7) | 974 (73.2) |
| Platelet | 60 (5.3) | 78 (6.6) | 67 (5.8) | 89 (7.0) | 98 (7.4) | 107 (7.7) | 73 (5.5) |
| Amount of blood transfusion, mean ± SD |  |  |  |  |  |  |  |
| Red blood cells* | 6.4 ± 6.2 | 6.6 ± 6.0 | 6.3 ± 5.8 | 6.8 ± 7.2 | 6.5 ± 5.5 | 6.7 ± 6.7 | 6.0 ± 5.0 |
| Fresh frozen plasma† | 7.1 ± 7.9 | 6.9 ± 6.7 | 7.0 ± 7.8 | 7.6 ± 9.1 | 7.3 ± 7.9 | 7.7 ± 10.1 | 6.7 ± 7.5 |
| Platelet‡ | 20.3 ± 17.9 | 20.8 ± 15.2 | 22.3 ± 16.9 | 24.9 ± 18.6 | 22.9 ± 17.8 | 24.1 ± 17.5 | 23.2 ± 11.9 |
| Hemostatic intervention, n (% of severe PPH managed in non-DPC facilities) |  |  |  |  |  |  |  |
| Intrauterine balloon tamponade§ |  | 15 (1.3) | 49 (4.2) | 116 (9.1) | 155 (11.8) | 191 (13.7) | 195 (14.7) |
| Arterial embolization | – (–) | 13 (1.1) | 17 (1.5) | 18 (1.4) | 19 (1.4) | 27 (1.9) | 16 (1.2) |
| Hysterectomy | 76 (6.7) | 83 (7.1) | 84 (7.2) | 92 (7.2) | 77 (5.8) | 86 (6.2) | 62 (4.7) |
| Non-DPC and DPC facilities |  |  |  |  |  |  |  |
| Cases, n (% of severe PPH) | 123 (3.4) | 150 (3.7) | 169 (4.0) | 153 (3.4) | 169 (3.5) | 175 (3.5) | 166 (3.3) |
| Frequency of blood transfusion,  n (% of severe PPH managed in DPC and non-DPC facilities) || |  |  |  |  |  |  |  |
| Red blood cells | 123 (100) | 150 (100) | 168 (99.4) | 153 (100) | 169 (100) | 175 (100) | 164 (98.8) |
| Fresh frozen plasma | 109 (88.6) | 132 (88.0) | 156 (92.3) | 145 (94.8) | 162 (95.9) | 164 (93.7) | 159 (95.8) |
| Platelet | 50 (40.7) | 48 (32.0) | 55 (32.5) | 42 (27.5) | 55 (32.5) | 52 (29.7) | 38 (22.9) |
| Amount of blood transfusion, mean ± SD |  |  |  |  |  |  |  |
| Red blood cells* | 16.1 ± 11.1 | 14.8 ± 9.5 | 15.7 ± 14.6 | 14.7 ± 10.9 | 14.4 ± 11.1 | 13.4 ± 8.6 | 12.3 ± 7.1 |
| Fresh frozen plasma† | 16.0 ± 13.2 | 14.9 ± 12.4 | 15.8 ± 19.4 | 15.2 ± 12.3 | 15.1 ± 13.5 | 14.5 ± 10.6 | 12.9 ± 9.3 |
| Platelet‡ | 24.6 ± 17.1 | 23.3 ± 15.0 | 23.4 ± 20.7 | 25.0 ± 22.3 | 24.6 ± 25.1 | 21.8 ± 16.8 | 23.9 ± 19.4 |
| Hemostatic intervention, n (% of severe PPH managed in DPC and non-DPC facilities) |  |  |  |  |  |  |  |
| Intrauterine balloon tamponade§ |  | 16 (10.7) | 26 (15.4) | 37 (24.2) | 50 (29.6) | 48 (27.4) | 58 (34.9) |
| Arterial embolization | * (*) | 28 (18.7) | 36 (21.3) | 31 (20.3) | 40 (23.7) | 31 (17.7) | 32 (19.3) |
| Hysterectomy | 15 (12.2) | 12 (8.0) | 12 (7.1) | 15 (9.8) | 10 (5.9) | 10 (5.7) | 13 (7.8) |

Due to the regulation of the Ministry of Health, Labour and Welfare, we cannot disclose data less than ten, and the related rows that counted backwards to reveal the data, thus showing as "-" and “*” in the results, respectively.

* Mean amount of transfused red blood cells in patients that performed transfusion.

† Mean amount of transfused fresh frozen plasma in patients that performed transfusion.

‡ Mean amount of transfused platelet in patients that performed transfusion.

§ Intrauterine balloon tamponade for abdominal hemorrhage were covered by insurance and thus counted since 2013.

|| In the cases of non-DPC and DPC facilities, patients took blood transfusion both in DPC and non-DPC facilities.
